# Role of Personal Protective Measures in Prevention of COVID-19 Spread Among Physicians in Bangladesh: a Multicenter Cross-Sectional Comparative Study

**DOI:** 10.1007/s42399-020-00471-1

**Published:** 2020-08-28

**Authors:** Md. Musab Khalil, Md Mashiul Alam, Mostafa Kamal Arefin, Mamunur Rashid Chowdhury, Muhammad Rezeul Huq, Joybaer Anam Chowdhury, Ahad Mahmud Khan

**Affiliations:** 1 Sheikh Russel National Gastroliver Institute and Hospital, Dhaka, Bangladesh; 2 Department of Cardiovascular Disease, Mayo Clinic, Rochester, MN USA; 3 Department of ENT & Head Neck Surgery, Dhaka Medical College, Dhaka, Bangladesh; 4 Department of Pediatric Ophthalmology & Strabismus, Ispahani Islamia Eye Institute and Hospital, Dhaka, Bangladesh; 5 Department of Clinical Neurology, National Institute of Neurosciences and Hospital, Dhaka, Bangladesh; 6 National Institute of Cardiovascular Diseases, Dhaka, Bangladesh; 7 Usher Institute, The University of Edinburgh, Edinburgh, UK

**Keywords:** COVID-19, Risk factors, Personal protective equipment, Healthcare workers, Physicians, Bangladesh

## Abstract

This study aims to determine the role of personal protective measures in the prevention of COVID-19 spread among the physicians working at different health facilities in Bangladesh. This hospital-based cross-sectional comparative study was conducted from May to June 2020. A total of 98 COVID-19 positive physicians and 92 COVID-19 negative physicians (physicians with no symptoms of COVID-19 or who tested negative) were enrolled. The questionnaire was adapted from a tool developed by the World Health Organization (WHO) for risk assessment and management of exposure of healthcare workers in the context of COVID-19. Data were collected from the respondents online using Google forms. There was no significant difference in baseline information between COVID-19 positive and COVID-19 negative physicians. The physicians, who were unaware of direct participation in COVID-19 patient care, had higher odds of being COVID-19 positive (OR = 4.018; CI: 1.532–10.535). Additionally, the physicians, who were unaware of the COVID-19 status while performing the aerosol-generated procedure (AGP), had a higher chance of being COVID-19 positive (OR = 2.522; CI: 1.020–6.233). Using face shields/goggles (OR = 0.437; CI:0.228–0.837) and regular decontamination of the patient’s surroundings (OR = 0.392; CI:0.176–0.873) while usual take care of patients and use of N95 masks while performing AGP (OR = 0.372; CI:0.159–0.873) had protective roles against COVID-19 among the physicians. The physicians who had reused the medical gown had two times more chances of being tested positive for COVID-19 than those who had not reused it (OR = 2.3; CI:1.251–4.259). The use of face shields/goggles and N95 masks and decontamination of the patient’s surroundings may give protection against COVID-19. Additionally, reusing medical gowns should be avoided as much as possible.

## Introduction

Coronavirus disease 2019 (COVID-19) has already affected millions of people with more than half-a-million deaths worldwide since the advent of SARS-CoV-2 in late 2019. Although some countries, e.g., China, Singapore, and South Korea, are forerunners to win this run against this deadly virus, this pandemic is still a high-level concern in most countries across the globe. At present, the infection rate and death toll are on the rise among South-Asian countries such as Bangladesh, India, and others [1]. Bangladesh has counted more than two hundred thousand infected people, along with numerous deaths [1, 2].

Health professionals are more vulnerable to COVID-19 than any other professionals as they have to work close to the patients [3]. The risk is higher among healthcare workers (HCWs) who are involved in the aerosol-generating procedure (AGP), such as noninvasive ventilation (NIV), high flow nasal cannula (HFNC), and endotracheal intubation [4]. HCWs may become a point of source to other non-COVID patients if they cannot be adequately contained.

Researchers in China reported 3387 infections among HCWs, which was 4.4% of all cases with 23 deaths [5]. According to the Italian National Institute of Health, approximately 17,000 HCWs were infected, which was 10% of Italy’s total cases [6]. Centers for Disease Control (CDC), in the USA, reported that more than 9200 HCWs were caught up with COVID-19 by April 2020 [7]. There is still no precise data about how many HCWs are infected with COVID-19 in Bangladesh. From a reliable source, it can be stipulated that as of July 18, 2020, about 3164 HCWs were affected [8].

Recent evidence shows that even asymptomatic persons can transmit COVID-19 with high efficiency, where conventional measures of protection such as face masks are insufficient [9, 10]. This virus may have an affinity to non-respiratory mucosal surfaces such as conjunctiva, which further limits the usefulness of face mask alone [11]. Another study showed that not only subclinical patients spread this virus, but also a person who had already recovered from acute illness can also shed a high amount of virus and thereby infect others [12]. This information warrants aggressive measures such as N95 masks, goggles/face shields, and protective gowns to ensure the safety of HCWs during patient care. Even with appropriate personal protective gear and proper hygiene, COVID-19 infection may occur [13]. HCWs had been forced to work without personal protective equipment (PPE), and legal actions had been taken against them for delaying to attend the patient due to a shortage of PPE [14]. The risk of transmission among healthcare professionals can be mitigated with appropriate precautions in health facilities [15–18]. Establishment of a clearer “zones of risk” and related protective measures can limit transmission in hospitals facing a limited supply of PPE [19].

In Bangladesh, physicians working in hospitals play a significant role in dealing with COVID-19 patients. A substantial number of physicians have already been diagnosed with COVID-19. Approximately 1200 physicians were infected, and 36 lost their lives [20]. It is time to take appropriate measures to prevent the spread of this grave disease among medical staff, especially among physicians. To better understand how to protect the physicians, we investigated the role of personal protective measures or PPE use in the prevention of COVID-19 spread among the physicians working at different health facilities in Bangladesh.

## Methods

### Study Design and Setting

A multicenter comparative cross-sectional study was conducted from May to June 2020 in different hospitals in Bangladesh. Some hospitals in Bangladesh were specialized for the treatment of COVID-19 positive patients only and were referred to COVID-dedicated hospitals. Suspected COVID-19 patients were referred from other hospitals to COVID-dedicated hospitals. However, sometimes non-COVID hospitals provided treatment to COVID-positive patients without knowing the COVID status of the patients.

### Study Participants

We collected the list of physicians from different hospitals whose reverse transcriptase-polymerase chain reaction (RT-PCR) test was positive. The controls were COVID-19 negative (having no symptoms of COVID-19 or tested negative) who worked in the same hospitals. In our study, we enrolled a total of 98 COVID-19 positive physicians and 92 COVID-19 negative physicians who work in different healthcare facilities and had known or unknown interactions with COVID-19 patients.

### Sampling Technique

First, we approached all COVID-19 positive physicians of the different hospitals for enrollment as per our list. We contacted the COVID-19 negative physicians and enrolled a nearly equal number of physicians from the same hospital.

### Data Collection

We used a predesigned structured questionnaire typed to collect data. It was divided into five sections: (i) physician’s information, (ii) physician’s interactions with COVID-19 patient, (iii) physician’s activities performed on COVID-19 in the healthcare facility, (iv) adherence to infection prevention and control (IPC) procedures during healthcare interactions, and (v) adherence to IPC measures when performing AGP. The questionnaire was adapted from a tool developed by the World Health Organization (WHO) for risk assessment and management of exposure of healthcare workers in the context of COVID-19. The WHO tool has four options to quantify the frequency with which the physicians had taken personal protective measures: “Always” means more than 95% of the time; “Most of the time” means 50 to under 95%; “occasionally” means 20 to under 50%; and “Rarely” means below 20% [21]. In this study, we considered “Always” or “Most of the time” to define taking proper protective measures for each item. We used Google form to collect data online from the respondents after obtaining consent.

### Data Analysis

We used Statistical Product and Service Solutions (SPSS) version 26 to analyze data. Categorical variables were analyzed by the Chi-square test or Fisher’s exact test as applicable. A *p* value of less than 0.05 was considered statistically significant. We also calculated the odds ratio (OR) with 95% confidence interval (CI) using contingency table and logistic regression where appropriate.

## Results

This study revealed that the mean age of physicians, who were sampled for this study, was 32.7 ± 5.4 years, and the age of physicians had no impact on the chance of being COVID-19 positive. Our study shows that physicians working in the ICU/CCU/OT complex had a slightly increased chance of getting infection, although the difference was not statistically significant [OR = 1.244, CI: 0.402–3.845). Male physicians (OR = 1.152; CI: 0.590–2.249) and formal training on PPE use (OR = 1.667; CI: 0.890–3.121) mildly increased the odds of being infected, which was not significant (Table 1).

**Table 1:** Baseline information of the participants

Traits	Covid-19 positive *n* = 98	Covid-19 negative *n* = 92	*p* value	OR (95% CI)
	*N* (%)	*N* (%)		
Age in years				
<35	30 (30.6)	21 (22.8)	0.226^a^	Reference
≥35	68 (69.4)	71 (77.2)		0.670 [0.350–1.283]
Mean ± SD	32.7 ± 5.4	32.5 ± 3.8	0.704^b^	
Sex				
Female	22 (22.4)	23 (25.0)	0.679^a^	Reference
Male	76 (77.6)	69 (75.0)		1.152 [0.590–2.249]
Working hospital				
Outside Dhaka	17 (17.3)	11 (12.0)	0.295^a^	Reference
Inside Dhaka	81 (82.7)	81 (88.0)		0.647 [0.285–1.467]
Place of work				
Inpatient	41 (41.8)	34 (37.0)	0.702^a^	Reference
Outpatient/triage	23 (23.5)	30 (32.6)		0.636 [0.313–1.291]
Emergency	17 (17.3)	15 (16.3)		0.940 [0.410–2.155]
ICU/CCU/OT complex	9 (9.2)	6 (6.5)		1.244 [0.402–3.845]
Tertiary care	8 (8.2)	7 (7.6)		0.948 [0.312–2.880]
Received formal training on PPE use				
No	63 (64.3)	69 (75.0)	0.109^a^	Reference
Yes	35 (35.7)	23 (25.0)		1.667 [0.890–3.121]

^a^Chi-square test; ^b^Independent samples *t* test; ^c^Fisher’s exact test

Table 2 depicts that physicians, who were unaware of any contact with COVID-19 patients, had lower odds of being COVID-19 positive (OR = 0.352; CI: 0.131–0.945). However, when asked about direct participation in COVID-19 patient care, such unawareness shows higher odds (OR = 4.018; CI: 1.532–10.535), and this association is statistically significant (*p* = 0.004). Physicians, who were unaware of the COVID-19 status while performing AGP, also had a higher chance of being COVID-19 positive (OR = 2.522; CI: 1.020–6.233). On the other hand, direct contact with contaminated fomites had no significant association with COVID-19 positivity.

**Table 2:** Exposure of the participants to the COVID-19 patients

Traits	Covid-19 positive *n* = 98	Covid-19 negative *n* = 92	*p* value	OR [95% CI]
	*N* (%)	*N* (%)		
Contact with COVID-19 patients				
Hospital environment	41 (41.8)	35 (38.0)	0.113^c^	Reference
Suspected COVID-19 patient or health worker	23 (23.5)	13 (14.1)		1.510 [0.668–3.416]
Confirmed COVID-19 patient or health worker	23 (23.5)	24 (26.1)		0.818 [0.395–1.695]
Community source	4 (4.1)	3 (3.3)		1.138 [0.238–5.435]
Unknown	7 (7.1)	17 (18.5)		0.352 [0.131–0.945]
Participated in direct COVID-19 patient care				
No	32 (32.7)	36 (39.1)	0.004^a^	Reference
Yes	41 (41.8)	49 (53.3)		0.941 [0.501–1.770]
Unknown	64 (25.5)	7 (7.6)		4.018 [1.532–10.535]
Performed aerosol-generating procedures on COVID-19 patient				
No	58 (59.2)	65 (70.7)	0.118^a^	Reference
Yes	22 (22.4)	19 (20.7)		1.298 [0.639–2.636]
Unknown	18 (18.4)	8 (8.7)		2.522 [1.020–6.233]
Direct contact with contaminated fomites				
No	30 (30.6)	40 (43.5)	0.185^a^	Reference
Yes	43 (43.9)	33 (35.9)		1.737 [0.902–3.347]
Unknown	25 (25.5)	18 (20.7)		1.754 [0.819–3.757]

^a^Chi-square test; ^c^Fisher’s exact test

We calculated the odds ratio of different protective measures taken by the physicians, which might have some role to prevent catching COVID-19 infection during usual patient care or while doing AGP (Tables 3 and 4). Face shields/goggles and regular decontamination of the patient’s surroundings had a protective role during usual patient care (OR = 0.437; CI: 0.228–0.837 and OR = 0.392; CI: 0.176–0.873, respectively). On the other hand, though not statistically significant, wearing PPE and proper handling of PPE might prevent catching this virus (OR = 0.146; CI: 0.018–1.212 and 0.570; CI: 0.286–1.137, respectively), while single-use gloves and wearing mask or disposable gown did not have a clear association among COVID-19 positive and COVID-19 negative patients. Proper hand hygiene during different situations while dealing with patients had mixed results, and none of them was statistically significant (Table 3).

**Table 3:** Protective measures taken by the participants during usual care of COVID-19 patients

Protective measures	Covid-19 positive *n* = 98	Covid-19 negative *n* = 92	*p* value	OR [95% CI]
	*N* (%)	*N* (%)		
Wore PPE (*n* = 186)	90 (92.8)	88 (98.9)	0.066^b^	0.146 [0.018–1.212]
Single-use gloves (*n* = 179)	81 (90.0)	80 (89.9)	0.980^a^	1.013 [0.382–2.682]
Medical/surgical mask (*n* = 181)	89 (96.7)	85 (95.5)	0.717^b^	1.396 [0.303–6.423]
Face-shield/goggles (*n* = 180)	55 (59.8)	68 (77.3)	0.012^a^	0.437 [0.228–0.837]
Disposable gown (*n* = 179)	71 (78.9)	69 (77.5)	0.825^a^	1.083 [0.533–2.203]
Proper “doning” and “doffing” of PPE (*n* = 173)	59 (68.6)	69 (79.3)	0.109^a^	0.570 [0.286–1.137]
Followed hand hygiene during patient care (*n* = 172)	78 (92.9)	83 (94.3)	0.695^a^	0.783 [0.230–2.670]
Followed HH during procedure (*n* = 164)	75 (97.4)	80 (92.0)	0.175^b^	3.281 [0.661–16.297]
Followed HH after body-fluid exposure (*n* = 154)	67 (91.8)	79 (97.5)	0.151^b^	0.283 [0.055–1.447]
Followed HH after touching fomites (*n* = 166)	76 (93.8)	77 (90.6)	0.438^a^	1.579 [0.494–5.045]
Decontaminated surroundings (*n* = 165)	58 (72.5)	74 (87.1)	0.019^a^	0.392 [0.176–0.873]

^a^Chi-square test; ^b^Fisher’s exact test

**Table 4:** Protective measures taken by the participants during the aerosol-generating procedure

Protective measures	Covid-19 positive *n* = 98	Covid-19 negative *n* = 92	*p* value	OR [95% CI]
	*N* (%)	*N* (%)		
Wore PPE (*n* = 130)	58 (92.1)	64 (95.5)	0.483^b^	0.544 [0.124–2.376]
Single-use gloves (*n* = 124)	53 (93)	64 (94.4)	0.702^b^	0.621 [0.133–2.899]
N95 Mask (*n* = 122)	36 (65.5)	56 (83.6)	0.021^a^	0.372 [0.159–0.873]
Face-shield/goggles (*n* = 122)	39 (70.9)	52 (77.6)	0.397^a^	0.702 [0.310–1.593]
Disposable gown (*n* = 123)	43 (78.2)	56 (82.4)	0.562^a^	0.768 [0.314–1.876]
Water-proof apron (*n* = 123)	19 (34.5)	35 (51.5)	0.060^a^	0.498 [0.239–1.034]
Proper “doning” and “doffing” of PPE (*n* = 126)	41 (70.7)	56 (82.4)	0.121^a^	0.517 [0.223–1.199]
Followed Hand Hygiene during patient care (*n* = 124)	54 (93.1)	62 (93.9)	0.850^a^	0.871 [0.208–3.671]
Followed HH during procedure (*n* = 122)	51 (91.1)	62 (93.9)	0.731^b^	0.658 [0.168–2.579]
Followed HH after touching fomites (*n* = 122)	48 (85.7)	60 (90.9)	0.370^a^	0.600 [0.195–1.847]
Decontaminated surroundings (*n* = 119)	35 (62.5)	47 (74.6)	0.155^a^	0.567 [0.259–1.243]

^a^Chi-square test; ^b^Fisher’s exact test

During AGP in COVID-19 patients, wearing the N95 mask was significantly associated with a low probability of COVID-19 infection (OR = 0.373; CI: 0.159–0.873). In contrast, wearing PPE, single-use gloves, protective face-shields/ goggles, disposable gown, water-proof apron, proper handling of PPE, proper hand-hygiene during different patient care, and decontamination of the surroundings of the patient decreased the chance of COVID-19 infection among physicians. However, none of these results were statistically significant (Table 4).

**Fig. 1:**
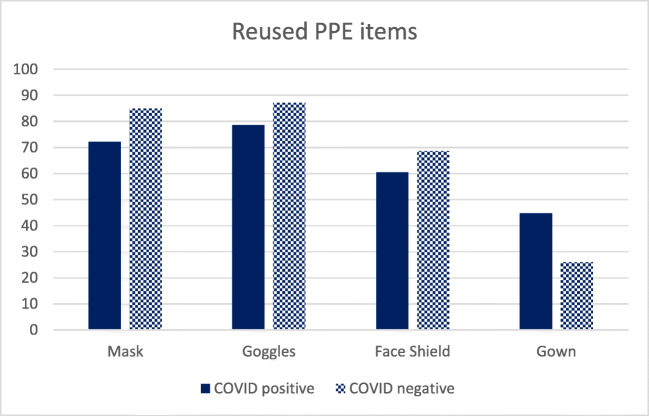
Pattern of reused PPE items among the participants

As most physicians had to reuse their PPE items, we also investigated the role of reusing PPE items in catching COVID-19 infection among physicians. Figure 1 shows that more physicians, who were not diagnosed with COVID-19, had reused masks, goggles, and face-shields than physicians who were tested COVID positive. However, these associations were not statistically significant (*p* > 0.05). In addition, physicians who had reused their protective gown had two times more chances to be tested positive in comparison with physicians who did not reuse their gown (OR = 2.3; CI:1.251–4.259; *p* = 0.007).

## Discussion

Risk factor assessment of COVID-19 among physicians is a timely issue. To date, large numbers of physicians have become infected while treating COVID-19 patients [20]. Bangladesh is one of the worst affected countries in terms of physicians being affected and deceased [22]. This study was conducted to determine the personal protective measure-related factors responsible for these large numbers of physicians being affected by COVID-19.

The mean age of the affected physicians was 32.7 ± 5.4 years. A study conducted on physicians in the USA showed that the median age of physicians being affected was 42 years [7]. Another study conducted in Wuhan, China, revealed that the mean age of the affected physicians was 37 years [23]. From this finding, it seems that the physicians in Bangladesh were affected at a relatively younger age. At Wuhan, relatively older-aged physicians were affected with COVID-19, and the age difference between infected and uninfected physicians was significant [23]. However, in our study, no statistically significant difference was found regarding age between these two groups. We found that male physicians had increased odds of being infected, although this was not statistically significant. This sex difference was not statistically significant in the study at Wuhan as well [23]. However, female physicians were affected with COVID-19 2.5 times more than their male colleagues in the USA [7].

This study revealed that the chances of becoming infected were higher among patients in the ICU than among inpatients, but the difference was not statistically significant. In contrast, Wuhan’s study showed that physicians working in the ICU had two times more chances of becoming infected than the general wards [23]. This difference could be due to the lower number of respondents working in the ICU in our study.

Formal PPE training did not have any significant impact in our study on being infected with COVID-19 or not. None of the physicians from Wuhan contacted COVID-19 infection after being adequately trained about PPE [24]. This contrasting picture in this study could be due to the lack of supervision and monitoring about how to use PPE after the physicians were trained to use PPE.

An interesting finding of our study was that the physicians, who were unaware of any contact with COVID-19 patients or who were unaware of the patient’s COVID-19 status during AGP, had a higher chance of being COVID-19 positive. This could be an asymptomatic or pre-symptomatic transmission of the SARS-CoV-2 virus through respiratory droplets [9]. This finding recommends taking appropriate protective measures during direct patient care and performing AGP until the physician is confident that the patient is not suffering from COVID-19, especially in this pandemic situation.

This study revealed that the proper use of face shields or goggles significantly protected the physicians from COVID-19. Using the face shield is also recommended by the WHO, especially during AGP [25]. This study also showed that decontaminated hospital surroundings also significantly increased infection rates among physicians. Lack of control of environmental decontaminants and inadequate infection prevention and control measures might have contributed to the infection. A proper implementation would mitigate this problem [26]. N95 masks provided a protective factor against COVID-19 among those who performed AGP. The WHO also recommends N95 mask use during performing AGP. An interesting finding was that physicians who reused gowns had significantly two times higher chances of becoming infected with COVID 19 than others. This result emphasizes the proper use and adequate supply of PPEs, which are of utmost importance for preventing infections among physicians.

The study had some limitations as well. As the physicians had to recall their events while filling up the questionnaire, there might be a chance of recall bias. Additionally, the sample size was small. We could not include physicians of all age groups. Furthermore, a large-scale study may be helpful to determine the actual reason behind the high rate of infections among physicians in Bangladesh.

## Conclusion

Despite the limitations, this study helped to understand the reasons behind the infection risks of healthcare providers. Proper use of the face shield, adequate decontamination of the patient’s surroundings during the usual patient encounter, and wearing the N95 mask and not reusing medical gowns during AGP could be the game changer.
